# Diagnoses and Treatment of Acquired Undescended Testes: A Review

**DOI:** 10.1097/MD.0000000000038812

**Published:** 2024-07-05

**Authors:** Ya-Long Ma, Ti-Xue Wang, Lin Feng, Chuan-Bing Hu, Jin-Song Sun, Chong-Fang Zhang, Bao-Hua Yu

**Affiliations:** a Department of Clinical Medicine, Jining Medical University, Jining, Shandong Province, China; b Department of Pediatric Surgery, Affiliated Hospital of Jining Medical University, Jining, Shandong Province, China.

**Keywords:** acquired undescended testes, diagnosis, etiology, treatment

## Abstract

Acquired undescended testes were once considered a sporadic disease. In recent years, reports suggest that they are not uncommon, with an incidence rate about 3 times that of congenital undescended testes. The etiology of acquired undescended testes remains inconclusive, clinical diagnostic standards are unclear, and treatment approaches are still controversial. There is ongoing debate about the mechanism of testicular ascent. The prevailing view is that acquired undescended testes occur due to the partial absorption of the gubernaculum, which forms part of the parietal peritoneum. The residual gubernacular fibers continuously pull on the spermatic cord, preventing the spermatic cord from elongating proportionately to somatic growth, leading to a re-ascent of the testis. Acquired undescended testes may increase the risk of testicular cancer, but this is still debated. The preferred treatment method is also controversial. However, surgical fixation has an immediate effect; no studies have proven that early surgery improves fertility in patients. The etiology of acquired undescended testes is closely related to the continuous pull of the residual gubernacular fibers on the spermatic cord, which prevents the cord from extending proportionately to body growth. There are no clear diagnostic standards for acquired undescended testes yet, and spontaneous descent is possible, so testicular fixation surgery may not be the preferred treatment method.

## 1. Introduction

Undescended testes (UDT) is a common reproductive disorder in male children, which can impair patients’ reproductive potential and increase their risk of testicular cancer.^[1]^ UDT can be classified into congenital undescended testes (cUDT) and acquired undescended testes (aUDT).^[2,3]^ Those who are born with UDT are cUDT patients. If UDT occurs in late childhood, it is called aUDT.^[4]^ aUDT refers to the undescended testes found due to the ascent of testes without groin surgeries. The testes of these patients were found in the scrotum in past physical examinations by experienced urological surgeons.^[5,6]^ So far, no conclusion has yet been reached on the causes of aUDT and the criteria for clinical diagnoses. Research on the treatment for aUDT has proved inconclusive.

## 2. Background information and epidemiological features

In the past, UDT was considered a congenital disorder that can result in impaired reproductive potential and an increased risk of testicular cancer in children. The risk was reduced by orchiopexy in the early stages of the UDT. However, recent literature suggests that UDT can occur not only congenitally but also in later childhood and is called ascending testes.^[7]^ In 1966, Villumsen and Zachau-Christiansen^[8]^ conducted a 3-year observation of 4300 boys born with regular testicular positions. They found that 87 testicles of 69 boys ascended above the scrotum on later examination, among which eleven were located higher than the external groin ring.^[9]^

About 310 boys treated with orchiopexy in 6 pediatric medical institutions in Germany were investigated regarding testicular position at birth and age at surgery. It was found that cUDT and aUDT were equally common in patients with known previous testicular positions. More than half of the testicular fixations performed after 1 year were due to aUDT. However, only 15% of physicians consider aUDT to be an indication of late surgery. aUDT is far more common than previously thought, occurring about 3 times more often than cUDT, and needs to be better recognized in clinical practice.^[10,11]^

As more and more scholars consider aUDT an independent disorder, more and more research on the difference between cUDT and aUDT.^[12,13]^ A study has shown subtle differences in hormone levels exist between the 2. Insulin-like peptide 3 (INSL3) produced by stromal cells plays a crucial role in the testes’ descent into the abdomen. It is believed to be a more sensitive parameter for detecting impaired interstitial cell function compared with testosterone.^[14–17]^ Anti-Mullerian hormone (AMH) produced by Sertoli cells relates to testicular volume and semen concentration.^[18]^ AMH is considered a marker of male Sertoli cell function. The 2 indexes detected differences between cUDT and aUDT. The function of interstitial cells detected by INSL3 showed the same impairment in cUDT and aUDT. The degree of Sertoli cell function impairment evaluated by AMH in cUDT was higher than that in aUDT.^[19]^

Additionally, there are also structural differences between the 2. cUDT mainly locates at the proximal end of the external inguinal ring with vaginal process deformities and epididymis malformations. However, aUDT usually appears at the distal end of the external inguinal ring and most commonly at the subcutaneous inguinal ring with partial vaginal process deformities and no epididymis malformations.^[20–22]^

In 1964, Scorer reported that the incidence of UDT in newborns weighing at least 2500 kg was 2.8%. However, the rate of orchiopexy performance was several times higher than the rate of UDT. Follow-up studies showed that many patients were diagnosed and treated in childhood, and these patients’ testicles were precisely located in the scrotum before. Therefore, it was believed that aUDT played an essential role in the higher rate of orchiopexy.^[23]^ Dinkelbach and Lehnick retrospectively analyzed 151 cases of orchiectomy due to aUDT.^[24]^ They recorded the last time testes were found in the scrotum and the time of orchiopexy and estimated the time of testicular ascent with the average value of the 2. They found that testicular ascent occurred between 1 and 14 years, mostly occurring before adolescence. Therefore, boys are recommended to have their testes regularly checked before the age of puberty.^[25,26]^

## 3. Etiology

**Figure 1.**
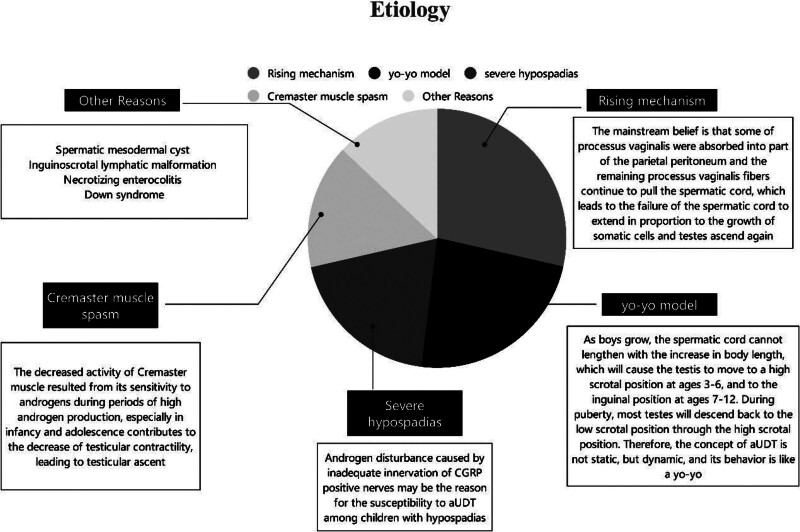
Associated etiology of acquired undescended testes.

The etiology of aUDT is manifold (Fig. 1). The mechanism of ascending testes remains a controversial issue. The mainstream belief is that some of the processus vaginalis were absorbed into part of the parietal peritoneum, and the remaining processus vaginalis fibers continue to pull the spermatic cord. This leads to the failure of the spermatic cord to extend in proportion to the growth of somatic cells, and testes ascend again.^[9,27]^

However, an increasing number of studies suggest that a critical factor in the pathogenesis of aUDT is the relative insufficiency of spermatic cord length.^[28]^ The scrotum gradually moves away from the inguinal region as the child grows. Suppose the length of the spermatic cord is insufficient. In that case, the testis may remain stationary, unable to maintain a stable position in the scrotum, moving from a high scrotal position to the inguinal region.^[6]^ Hack and Goede^[29]^ proposed the yo-yo model, which helps explain some hitherto unexplained phenomena of aUDT. As boys grow, the spermatic cord cannot lengthen with the increase in body length, which will cause the testis to move to a high scrotal position at age 3 to 6 and to the inguinal position at age 7 to 12. During puberty, most testes will descend back to the low scrotal position through the high scrotal position. Therefore, aUDT is not static but dynamic, and its behavior is like a yo-yo.^[29]^

Boys with severe hypospadias have a significantly higher risk of developing aUDT as a complication.^[30]^ The research of Itesako and Nara suggested that the risk of aUDT as a complication was directly linked to the severity of hypospadias.^[31]^ However, the reasons remained unknown. According to Nazir et al, androgen disturbance caused by inadequate innervation of CGRP-positive nerves may be the reason for the susceptibility to aUDT among children with hypospadias. For boys with hypospadias, especially those with severe hypospadias, serious attention should be paid to changes in their testicular position.^[32,33]^

Besides, Cremaster muscle spasms may be 1 of the possible causes of aUDT among children with cerebral palsy. Nevertheless, its causes among ordinary boys are still unknown. The decreased activity of the Cremaster muscle resulting from its sensitivity to androgens during periods of high androgen production, especially in infancy and adolescence, contributes to the decrease of testicular contractility, leading to testicular ascent.^[34]^ Others believe that the remaining fibrous cords from the partial processus vaginalis deformity exist in the depth of spermatic fascia, leading to the failure of umbilical cord extension. Impaired umbilical cord extension is widespread in children with Down syndrome, who also have a higher incidence of aUDT.^[35]^

Other rare factors may also cause aUDT. Vaos and Zavras reported the testicular ascent in a 2-year-old boy who was found to have a spermatic mesodermal cyst (MCSC) due to a mass in his groin.^[36]^ Therefore, MCSC may be responsible for developing aUDT. MCSC narrows the inguinal canal excessively and impedes the standard descent path of the testicle, which may result in ascending testes in the scrotum. Uebayashi and Ohno reported a case of swelling of the groin area and scrotum caused by lymphatic malformation (LM).^[37]^ Eppikajutsuto, a traditional Japanese herbal medicine, was used in the treatment. It was found that while TJ28 reduced the size of LM, it enabled the retraction of the left testicle into the groin area. As a result, the treatment of inguinoscrotal LM should consider the possibility of the development of aUDT during the treatment. Ademaj et al^[38]^ reported a case of aUDT caused by postoperative necrotizing enterocolitis (NEC) adhesions. NEC is the most common cause of intraperitoneal adhesions among newborns. The testicles of patients, in this case, were born in the scrotum. These patients received NEC surgeries when they were newborn babies. Their testicles were not in the scrotum on their return visits. Ultrasonography suggested that the testicles had ascended into the abdominal cavity. Further laparoscopic exploration found that the lower end of the testicle adhered to the sigmoid colon. It was concluded from this case that the testes of the newborn are very likely to return to the abdominal cavity due to postoperative adhesions after abdominal surgeries, thus leading to aUDT.

## 4. Complications

The risk of testicular cancer with aUDT is still in dispute. However, there are other disorders induced by aUDT.

### 4.1. Testicular microlithiasis

The research of Goede and Hack conducted follow-ups among children with aUDT and screened for testicular microlithiasis (TM) by ultrasound.^[39]^ TM is defined as shadowless echogenic focuses within the testicular parenchyma. Typical microlithiasis shows 5 or more echogenic focuses in 1 or both testicles. In comparison, limited microlithiasis shows 5 or fewer echogenic focuses. Results indicated that the incidence of TM in children with aUDT was 2.8%.

### 4.2. Varicocele

Meij-de Vries and den Bakker reported a high incidence of intratesticular varicoceles (ITV) among children who suffered from aUDT after orchiopexy.^[40]^ The testicular volume of ITV patients was generally smaller. However, whether ITV was induced by orchiopexy or aUDT needs further research.

## 5. Diagnostic criteria

The diagnostic criteria for aUDT are in dispute. To distinguish it from cUDT, the following 3 criteria are recommended.

1. A reliable medical history supports the fact that the testicle was once at the bottom of the scrotum.
2. Undescended testes cannot be put back into the scrotum by experienced urologists, even if the patients are under anesthesia.
3. It is impossible to find factors that cause the ascent of testicles on the same side, such as inguinal surgeries, trauma, or inflammation.^[9]^

## 6. Treatment

The top choice of treatment for aUDT remains a controversial issue. Current treatments include orchiopexy, spontaneous testicular descent, and hormone therapy.

Research by Sijstermans and Hack assessed the possibility of spontaneous testicular descent among pre-adolescent boys with aUDT.^[41]^ According to Tanner scales, adolescence was classified into early, middle, and late stages. Early adolescence includes the G2 stage with 4 to 9 mL testicular volume. Middle adolescence includes the G3 stage with a testicular volume of 10 mL and the G4 stage with a testicular volume of eleven to fifteen mL. Late adolescence includes the G5 stage with a testicular volume over fifteen mL.^[41]^ About 129 children with aUDT were followed up to the Tanner G3 stage, and their testicular position and volume were regularly checked. Orchiopexy was performed on testes that failed to descend spontaneously after the Tanner G3 stage. About 98 of the children were observed to have spontaneous testicular descent. Among them, 70 happened at Tanner G2 stage, 26 at G3, and 2 at G5 when prepared for orchiopexy. Conservative treatment until the Tanner G3 stage can help more than half of patients avoid orchiopexy, and the volume of testes descending spontaneously is closer to that of a normal 1.^[42]^

The role of human chorionic gonadotropin (HCG) is highly controversial in the treatment of aUDT. The study of Barthold and Gonzalez indicated that only 50% of children with aUDT were sensitive to HCG and showed testicular descent after the use of HCG.^[43]^ However, 1 research conducted by Meijer and Hack found that all 11 children with aUDT showed testicular descent after the use of HCG.^[44]^ Ten of them were reported symmetrical testes of average size. Due to inadequate data in available research, more extensive data are needed for a more accurate evaluation of results.^[11,45]^

Orchiopexy is a common choice of treatment for aUDT,^[22,46]^ which may explain why there is a high incidence of ORP performance in childhood in most countries.^[47]^ Despite its sound instant effects, no studies have proved that early ORP treatment can improve patients’ fertility.^[48]^ Recent research suggests that aUDT usually lies near the subcutaneous ring in the groin. Some testes manage to stand the strain of the remaining fibrous cords from the processus vaginalis in the spermatic cord and descend spontaneously under regular hormonal stimulation during adolescence. The volume of testes of patients with spontaneous testicular descent shows subtle differences from that of an average man. Moreover, such children are less likely to suffer from epididymal abnormalities.^[49]^ The effect of HCG in the treatment of aUDT needs further research.^[50]^ Previous literature has not demonstrated the effect of luteinizing hormone-releasing hormone in treating aUDT.^[12]^

**Figure 2.**
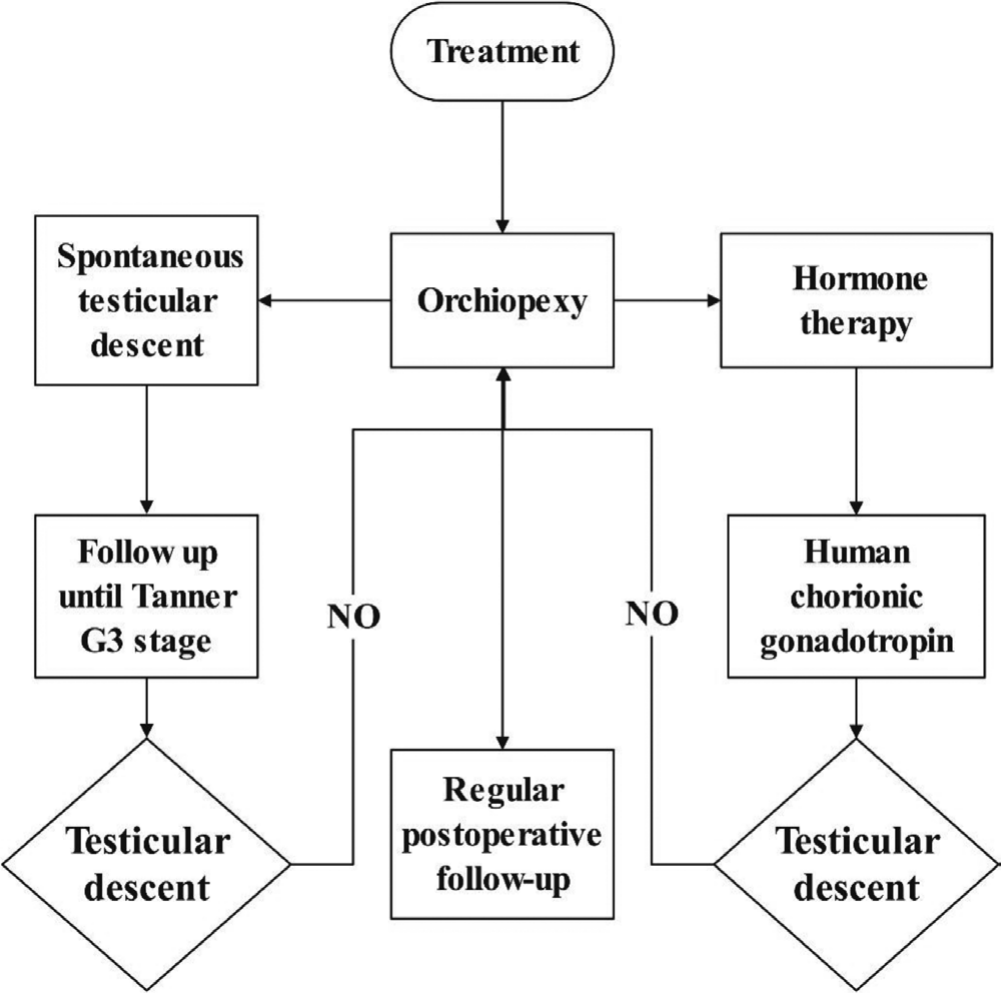
Treatment associated with acquired cryptorchidism.

aUDT may undergo histopathologic changes similar to cUDT,^[51]^ such as decreased size and reduced spermatogonia, which may affect reproductive function. Although aUDT has a high probability of spontaneous descent by puberty, aUDT has histopathologic changes similar to those of cUDT, which may affect reproductive function.^[52]^ The efficacy of hormonal therapy for aUDT is unclear. Most scholars believe that surgical intervention should be performed immediately after the definitive diagnosis of aUDT, and testicular descent fixation should be performed. However, a systematic evaluation of the long-term effects of testicular descent fixation in aUDT, such as changes in testicular volume and spermatogonial cell counts, is still lacking. In summary, surgical treatment remains the mainstay of therapy for aUDT. However, further studies are needed to examine natural developmental outcomes after the natural descent of aUDT and to analyze long-term outcomes after performing testicular fixation (Fig. 2).^[53]^

## 7. Conclusion

The etiology of aUDT still needs to be completed, and there are few clear diagnostic criteria. Early testicular immobilization is often used as the mainstay of treatment. However, so far, there are no long-term follow-up studies to justify this treatment, and aUDT has a clear and persistent trend of spontaneous descent with age, with the majority of children’s testes reaching a normal scrotal position around puberty. The increase in luteinizing hormone and testosterone levels at puberty may account for the spontaneous descent of the testes. The increase in luteinizing hormone and testosterone levels during puberty may be the cause of spontaneous testicular decline. Therefore, conservative treatment can be performed before puberty and observed until TannerG3, after which testicular immobilization can be performed on testes that have not spontaneously descended. However, a large sample of studies still needs to confirm the development of naturally descending testes. According to the literature, testes located in the inguinal region tend to have more difficulty spontaneously descending into the scrotum than testes located above the scrotum, so aUDT located in the inguinal region or higher should be surgically intervened as early as possible. The use of HCG in the treatment of aUDT is still highly controversial, and longitudinal studies with larger sample sizes are needed to clarify its therapeutic effects.

## Author contributions

**Conceptualization:** Ya-Long Ma, Bao-Hua Yu.

**Data curation:** Ya-Long Ma.

**Methodology:** Ya-Long Ma, Bao-Hua Yu.

**Writing – original draft:** Ya-Long Ma.

**Validation:** Ti-Xue Wang, Chuan-Bing Hu, Jin-Song Sun, Chong-Fang Zhang.

**Investigation:** Lin Feng, Chuan-Bing Hu.

**Visualization:** Lin Feng.

**Writing – review & editing:** Bao-Hua Yu.
